# Concurrent Acute Myeloid Leukemia and Autoimmune Hemolytic Anemia: Management Challenges and Clinical Insights: A Rare Case Report

**DOI:** 10.1002/ccr3.9620

**Published:** 2024-11-29

**Authors:** Mais Musleh, Qossay Alhusein

**Affiliations:** ^1^ Department of Hematology, Faculty of Medicine Al Assad University Hospital Damascus Syria; ^2^ Department of Hematology, Faculty of Medicine Al‐Mouwassat University Hospital Damascus Syria

**Keywords:** AIHA, AML, diagnostic challenges, rare, Syria, therapeutic management

## Abstract

The simultaneous occurrence of acute myeloid leukemia (AML) and autoimmune hemolytic anemia (AIHA) is exceedingly rare, with an incidence of less than 1%. We report the case of a 50–year–old patient newly diagnosed with this uncommon combination. This case underscores the complexity and infrequency of this dual diagnosis, highlighting the diagnostic and therapeutic challenges it presents.

**Abbreviations**
AIHA: Autoimmune hemolytic anemia
AML: Acute myeloid leukemia

**Summary**

- The simultaneous occurrence of acute myeloid leukemia (AML) and autoimmune hemolytic anemia (AIHA) is exceedingly rare, with an incidence of < 1%.
- We report the case of a 50‐year‐old patient newly diagnosed with this uncommon combination.
- This case underscores the complexity and infrequency of this dual diagnosis, highlighting the diagnostic and therapeutic challenges it presents.

## 1 Introduction

Acute myeloid leukemia (AML) is a heterogeneous hematologic malignancy characterized by the clonal proliferation of myeloid precursors [1]. In contrast, autoimmune hemolytic anemia (AIHA) is a rare autoimmune disorder wherein the immune system erroneously targets and destroys erythrocytes, resulting in hemolysis and subsequent anemia. AIHA is well‐documented in patients with lymphoid neoplasms; however, its prevalence is notably lower in individuals with other neoplasms such as chronic myeloid leukemia, myelodysplastic syndrome, AML, or myeloproliferative disorders transformed to acute myelocytic leukemia [2, 3, 4, 5]. The incidence of AIHA in patients with AML has been infrequently reported in the literature [6, 7, 8].

In this report, we describe a rare case involving a 50‐year‐old patient simultaneously diagnosed with AML and AIHA. This case underscores the critical importance of meticulous diagnostic evaluation and the inherent complexities in managing patients with concurrent hematologic pathologies. It emphasizes the necessity for individualized therapeutic strategies to effectively address the coexistent conditions of AML and AIHA, highlighting the imperative for personalized treatment approaches in such intricate clinical scenarios.

## 2 Case History/Examinations

A 50‐year‐old male presented with generalized fatigue, significant weight loss, fever, and severe jaundice, evidenced by scleral icterus and dark urine. His medical history included hypothyroidism, managed with L‐thyroxine, and type 2 diabetes, controlled with metformin. Physical examination revealed no hepatosplenomegaly or lymphadenopathy.

**FIGURE 1:**
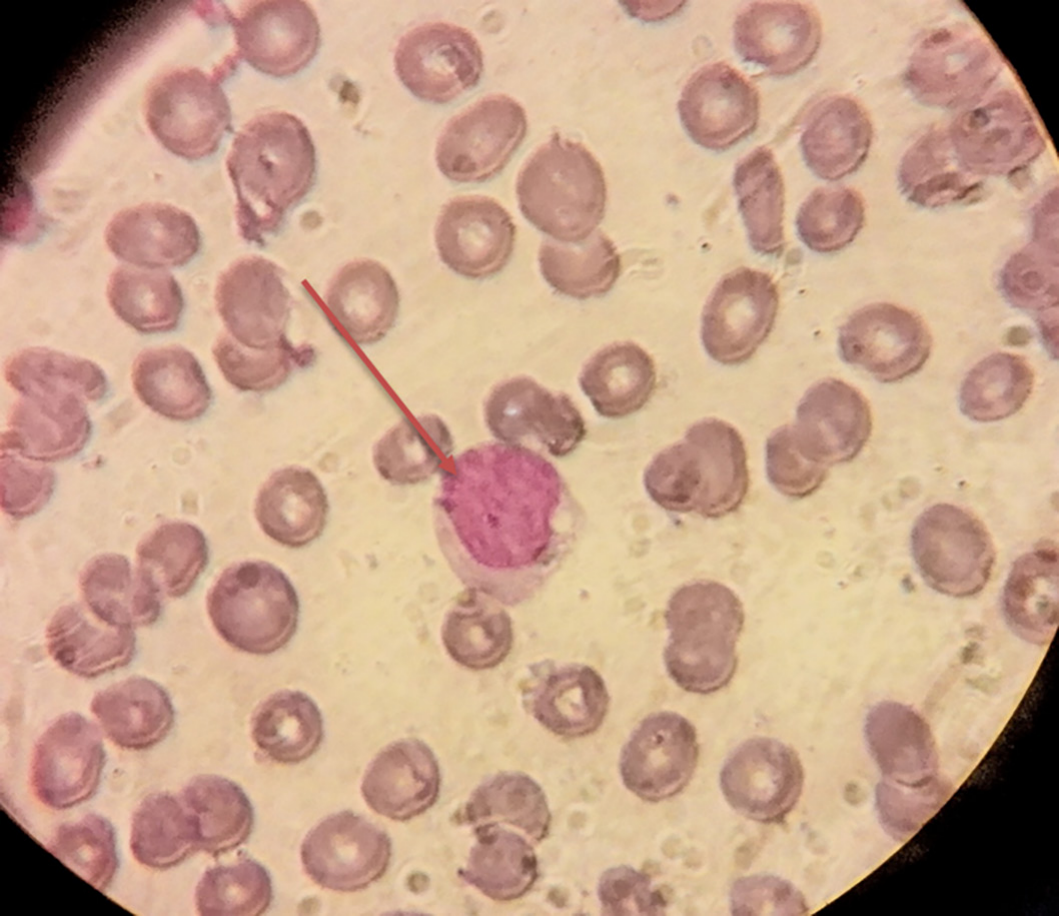
Peripheral blood smear showing myeloid blast cells, as referred with red arrow (Wright stain, 100× magnification).

**FIGURE 2:**
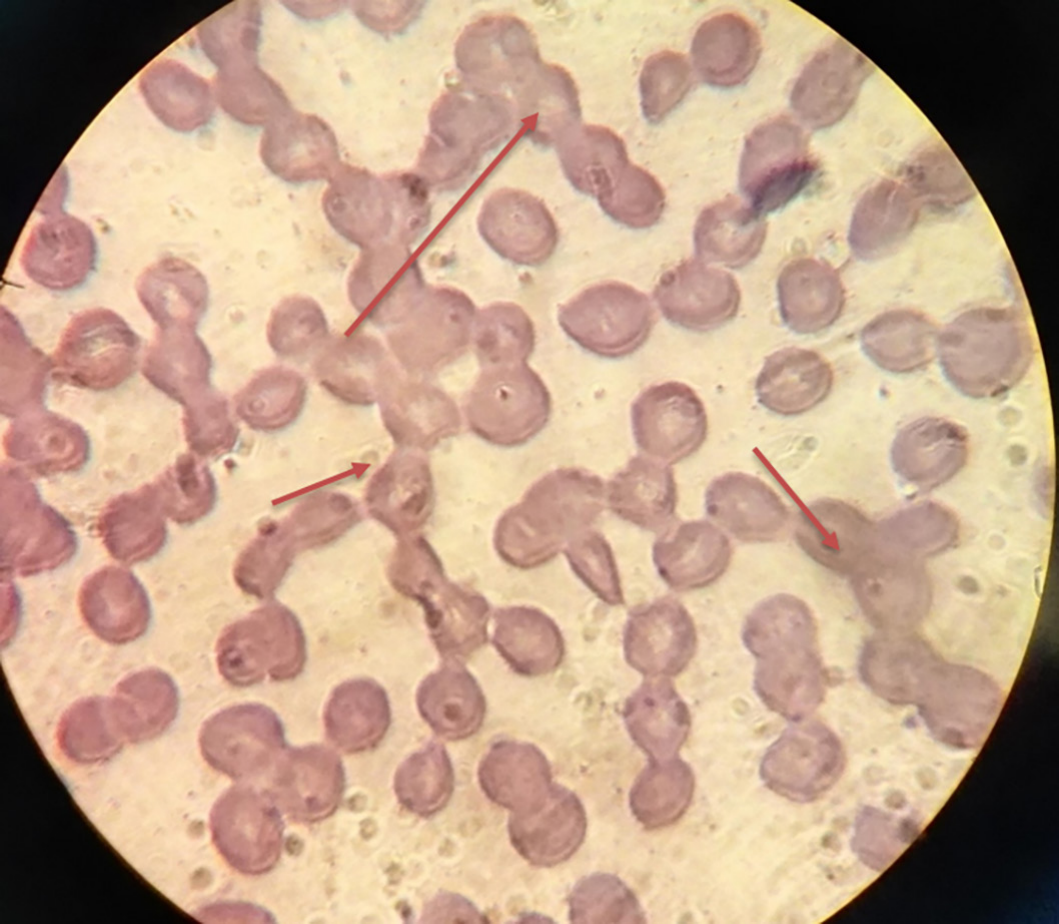
Peripheral blood smear revealing the presence of rouleaux in the red blood cells, as referred with red arrow (wright stain ×100).

**FIGURE 3:**
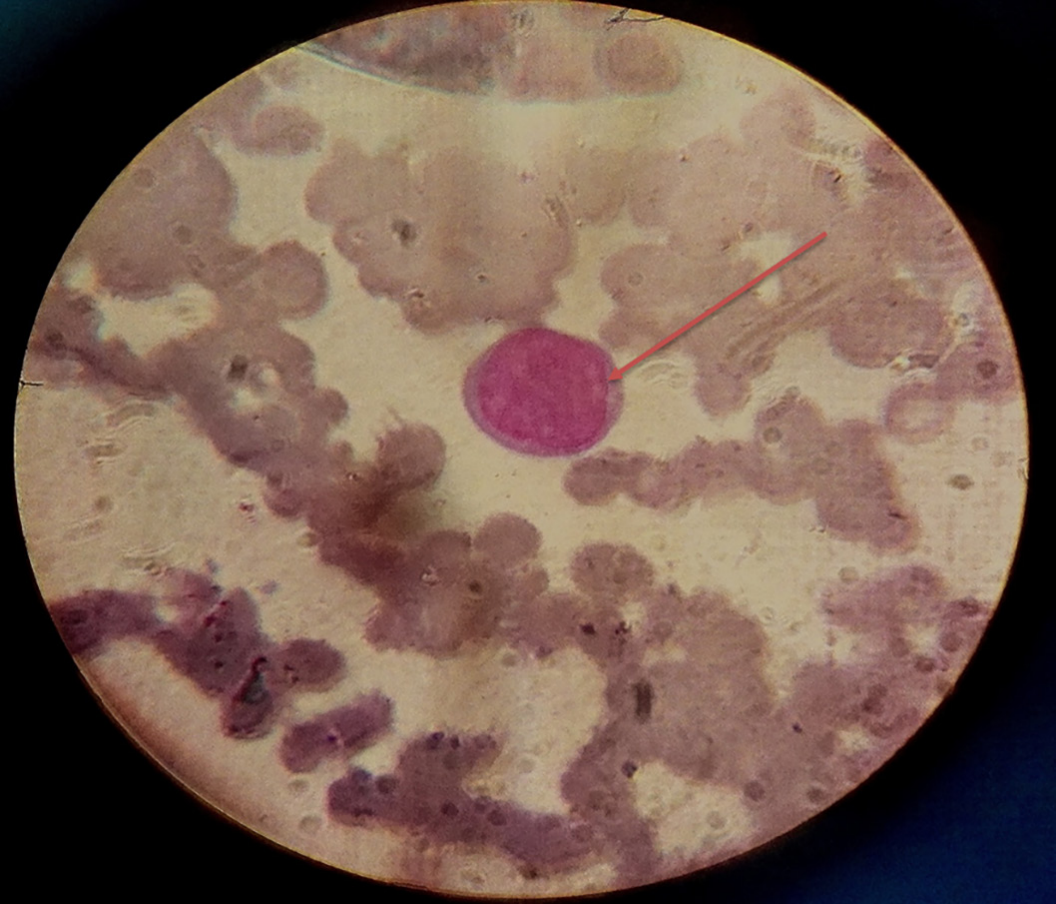
Bone marrow aspiration revealed infiltration of blast myeloid cells in bone marrow, as referred with red arrow (wright stain ×100).

**FIGURE 4:**
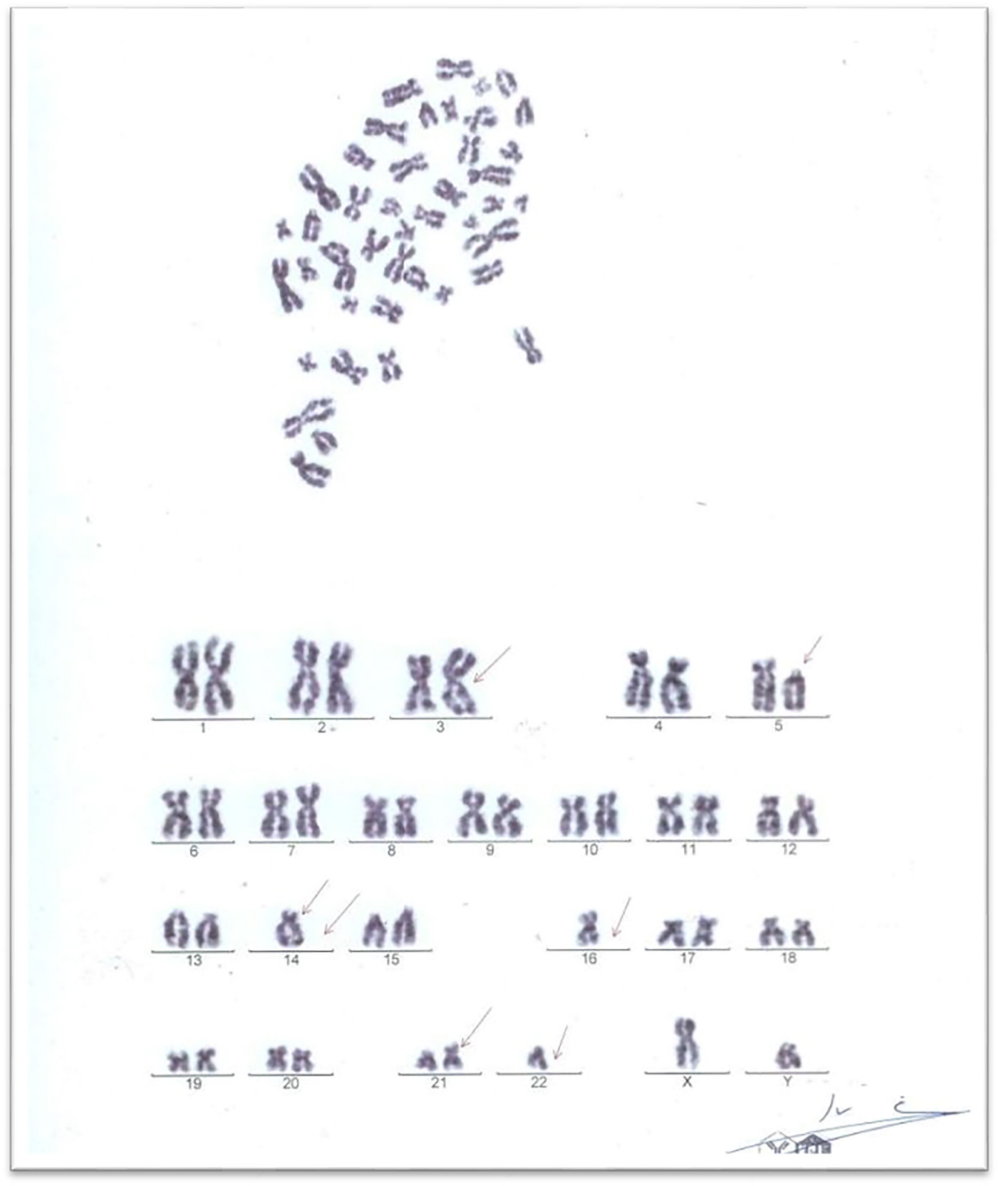
The results of cytogenetic analyses, which reveal abnormalities in the chromosomes.

Initial laboratory investigations indicated a white blood cell (WBC) count of 4.3 × 10^9^/L, hemoglobin (Hb) level of 5.7 g/dL, and platelet count of 55 × 10^9^/L. A peripheral blood smear showed 15% blasts with the presence of rouleaux in the red blood cells (Figures 1 and 2), Bone marrow aspiration confirmed infiltration by myeloid blasts (Figure 3). Immunophenotyping revealed that 40% of the myeloblasts expressed CD34, CD13, CD33, CD117, HLA‐DR, CD14, CD16, and CD64, confirming a diagnosis of AML according to the FAB classification. Cytogenetic analysis revealed a complex karyotype (43, XY t(3;5;14;21) del 14, 16, 22), as shown in Figure 4, categorizing the patient as adverse risk according to the ELN 2022 risk classification by genetics [9].

The workup for jaundice revealed indirect hyperbilirubinemia, decreased plasma haptoglobin [10 mg/dL (normal: 41–165 mg/dL)], and elevated lactate dehydrogenase (LDH) at 2300 U/L, with a positive +3 for direct Coombs' test (IgG). The reticulocyte count was 0.5%, attributed to bone marrow infiltration by blast cells.

The patient was administered prednisolone 1 mg/kg for a week, which improved Hb levels to 10.6 g/dL and stabilized bilirubin and LDH levels. This stabilization allowed for the initiation of induction chemotherapy (7 + 3) with cytarabine 200 mg/m^2^ as a continuous infusion for 7 days and daunorubicin 90 mg/m^2^ for 3 days, along with prednisolone at 1 mg/kg for 4 weeks, followed by a slowly tapering dose while Hb level has stabilized and markers of hemolysis are normal.

## 3 Differential Diagnosis

Jaundice and indirect hyperbilirubinemia may result from hemolysis related to liver dysfunction. Nevertheless, the absence of hepatosplenomegaly and normal hepatic enzyme levels makes hepatic infiltration or primary hepatic pathology less likely. A liver biopsy could be considered if hepatomegaly or other liver function abnormalities were identified.

Additionally, severe viral infections, such as those caused by Epstein–Barr virus (EBV) or cytomegalovirus (CMV), can occasionally present with cytopenias, hemolysis, and jaundice, mimicking hematologic malignancies. However, the identification of myeloid blasts and complex cytogenetic abnormalities in this patient's profile strongly supports a diagnosis of AML over infectious or inflammatory etiologies.

## 4 Conclusion and Results

Following induction therapy, the patient's condition improved significantly, with laboratory results showing a WBC count of 4.0 × 10^9^/L, neutrophils at 60%, lymphocytes at 40%, hemoglobin level of 10.5 g/dL, and platelet count of 100 × 10^9^/L. Bilirubin and LDH levels also stabilized, and bone marrow aspiration confirmed complete remission with < 5% blasts. The patient subsequently underwent re‐induction therapy with the 5 + 2 regimen, which included cytarabine 200 mg/m^2^ as a continuous infusion over 5 days and daunorubicin 90 mg/m^2^ for 2 days. This was followed by consolidation chemotherapy with high‐dose cytarabine at 3 g/m^2^ for one cycle.

Unfortunately, the patient's condition worsened during treatment, characterized by the onset of pancytopenia, with a white blood cell count of 0.7 × 10^9^/L, hemoglobin level of 9 g/dL, and a platelet count of 15,000/μL with a negative for direct Coombs' test. The patient subsequently developed severe neutropenic fever, ultimately leading to their demise.

## 5 Discussion

Acute myeloid leukemia is a complex hematologic malignancy characterized by the abnormal growth of myeloid precursor cells. In contrast, AIHA is a rare autoimmune disorder where the body targets and destroys its own red blood cells [1, 2]. Our patient presented with typical symptoms of AML and also exhibited features suggestive of AIHA.

The diagnosis of AIHA in this patient was primarily established through a positive direct anti‐globulin test in a normochromic normocytic anemia context, with the presence of nucleated red blood cells in the peripheral blood. Similarly, the diagnosis of acute leukemia was confirmed by identifying over 20% blasts in the bone marrow, which were characterized as myeloid in origin by positive CD13, CD33, and MPO markers on flow cytometry [10, 11].

Managing concurrent AML and AIHA presents significant challenges due to the distinct treatment approaches required for each condition. Corticosteroids, which are commonly used in AIHA management, are effective in approximately 70%–80% of cases, with most patients demonstrating an initial therapeutic response within 2–3 weeks. Once hemoglobin levels stabilize and markers of hemolysis normalize or show significant improvement—typically within this period—a gradual tapering of corticosteroids can be initiated [12, 13].

The standard induction therapy for AML typically involves intensive chemotherapy with cytarabine and an anthracycline, which are not known to induce hemolysis. Unlike agents such as fludarabine, which can be associated with immune‐mediated hemolysis. These chemotherapy regimens are aimed to achieve complete remission [14, 15, 16].

In managing our patient, our treatment approach was carefully designed to balance the requirements of both conditions. We started with prednisolone to control AIHA, followed by induction chemotherapy to target AML. This strategy led to an improvement in the patient's condition, highlighting the critical importance of a nuanced and adaptive treatment plan in managing such complex and concurrent diagnoses.

Initially, our combined treatment approach resulted in a temporary remission of both AML and AIHA. However, the patient's long‐term prognosis remained guarded due to the high likelihood of AML relapse and the potential recurrence of AIHA. AIHA significantly increases the risk of myeloid malignancies, elevating the risk of AML eightfold [17]. Unfortunately, severe neutropenic fever—a serious complication of intensive chemotherapy—ultimately led to the patient's death 3 months after diagnosis.

The underlying mechanisms behind the development of AIHA alongside de novo AML remain unclear. Some theories suggest antibodies targeting tumor antigens expressed on malignant erythroblasts, while others propose dysregulation in immunologic regulatory cells such as suppressor T cells, contributing to autoimmune reactions [5].

The coexistence of AML and AIHA is exceptionally rare, typically more associated with lymphoid malignancies than myeloid disorders. This case underscores the intricate management challenges presented by concurrent hematologic malignancies and autoimmune conditions, emphasizing the need for thorough diagnostic evaluation and personalized treatment strategies. Continued research aimed at elucidating the underlying pathophysiology and advancing therapeutic options is crucial for improving outcomes in this complex clinical scenario. Such efforts could lead to better understanding and management of patients facing this rare and challenging dual diagnosis, ultimately enhancing their prognosis and quality of life.

## Author Contributions

**Mais Musleh:** conceptualization, data curation, formal analysis, resources, software, validation, writing – original draft, writing – review and editing. **Qossay Alhusein:** conceptualization, project administration, supervision.

## Ethics Statement

The authors have nothing to report.

## Consent

Written informed consent was obtained from the patient for publishing this case report and any accompanying images. A copy of the written consent is available for review by the Editor‐in‐Chief of this journal on request.

## Conflicts of Interest

The authors declare no conflicts of interest.

## Guarantor

Qossay ALHUSEIN is the guarantor of this work.

## Data Availability

All data (of the patient) generated during this study are included in this published article.
